# Supplementary material for: Safety of Vaccines against SARS-CoV-2 among Polish Patients with Multiple Sclerosis Treated with Disease-Modifying Therapies
Source: Vaccines (Basel). 2022 May 12;10(5):763. doi: 10.3390/vaccines10050763 (PMC9147677; doi:10.3390/vaccines10050763)
Supplement: Supplementary file 1 [file vaccines-10-00763-s001.zip › vaccines-1700745-supplementary.pdf]

Supplementary Materials:

Vaccination against SARS-CoV-2 among individuals with multiple sclerosis. The questionnaire.

Date:

MS Center:

Neurologist performing the examination:

Patients age:

Patients sex:

Height:

Weight:

Smoker: Yes/No

Comorbidities:

Disease course: RRMS, PPMS, SPMS

Current EDSS:

Disease duration (years):

DMT (at the time of vaccination):

Duration of DMTs use (years):

Was DMT continued as planned during vaccination (was treatment deleyed?):

Date of last DMT use:

Relapse up to 3 months prior to vaccination: YES / NO

VACCINATION AGAINST SARS-COV-2

1. Type of vaccination (underline the correct answer):
- Pfizer
- Astra Zeneca
- Moderna
- Johnson & Johnson
- Novovax
- Sinovac
- Sputnik V
- Other:……………………..

2. Date of vaccination:

- First dose:
- Second dose:

3. Side effects after the first dose : YES/NO

Type of side effects (underline the correct answer/answers):
1.Pain at the injection site 2. Skin changes at the injection site 3. Fever, chills, flu-like symptoms 4. Fatigue 5. Headache 6. Pain in muscles, joints 7. Diarrhea 8. Nausea, vomiting 9. Abdominal pain 10. Malaise 11 Anaphylactic reaction 12. Others:………………….

Severity of symptoms: Mild/Moderate/Severe

Resolution of symptoms: after 1 day/ after 2-3 days/ after 4-5 days/ after 6-7 days/ after 7 days/ ongoing

4. Side effects after second dose : YES/NO

Type of side effects (underline the correct answer/answers):
1.Pain at the injection site 2. Skin changes at the injection site 3. Fever, chills, flu-like symptoms 4. Fatigue 5. Headache 6. Pain in muscles, joints 7. Diarrhea 8. Nausea, vomiting 9. Abdominal pain 10. Malaise 11 Anaphylactic reaction 12. Others:………………….

Severity of symptoms: Mild/Moderate/Severe

Resolution of symptoms: after 1 day/ after 2-3 days/ after 4-5 days/ after 6-7 days/ after 7 days/ ongoing

5. Severe post-vaccination complications: YES / NO
Type of complication: 1. Cerebral venous sinus thrombosis 2. Ischemic stroke 3. Cerebral haemorrhage 4. G-B syndrome 5. Encephalitis 6. Myelitis 7. Encephalomyelitis 8. VII nerve palsy 9. Other cranial nerve palsy 10. Peripheral nerve deficiy 11. Acute myopathy 12. Other:……………………………..

If cerebral venous sinus thrombosis was present, name location:………………………………………

The presence of risk factors for thrombosis: YES / NO
If YES, list specific factors:………………………………………..

Was the patient hospitalizes due to severe post-vaccination complications?: YES / NO

If YES: >10 days/ <10 days
Laboratory abnormalities:
Treatment:

Did the patient recovered?: 1. Yes 2. Not yet 3. Permanent neurological deficits remaining 4. Died due to complications 5. Died due to other reason:………………………….

6. Deterioration in the course of multiple sclerosis beyond relaps : YES / NO
If YES:
How many day after the first dose?: 1-2 days/ 3-5 days/ 6-8 days/ >8 days
Duration of deterioration: 1-2 days/ 3-5 days/ 6-8 days/ >8 days

How many day after the second dose?: 1-2 days/ 3-5 days/ 6-8 days/ >8 days
Duration of deterioration: 1-2 days/ 3-5 days/ 6-8 days/ >8 days

7. Relapse : YES /NO

Relapse after the first dose: YES/NO

If yes, how many days after the first dose: 1-7days/ 7-14 days/ 14-21 days/ >21 days

Relapse after the first dose: YES/NO

If yes, how many days after the first dose: 1-7days/ 7-14 days/ 14-21 days/ >21 days

Methylprednisolone (i.v.) use during relapse: 1-yes , 2-no , 3- there was no relapse

If YES please note the total dose:

Oral corticosteroids use as continuation after i.v. therapy : 1-yes , 2-no , 3- N/A

If YES please note name of the drug, total dose and the date of using:

Oral corticosteroids use as treatment of relapse without i.v. therapy : 1-yes , 2-no , 3- N/A

If YES please note name of the drug, total dose and the date of using:

8. SARS-CoV-2 infection after the vaccination : YES / NO

Date of diagnosis of the infection:

PCR result: 1-positive, 2-negative, 3-no information/ not done

Immunological test: 1-positive, 2-negative, 3-no information/ not done

Presence of antibodies: 1-positive, 2-negative, 3-no information/ not done

Contact with an infected person: 1-yes, 2-do not recall, 3-no information
